# Advanced Experimental Technology, Theory and Numerical Methods in Concrete Materials

**DOI:** 10.3390/ma19214673

**Published:** 2026-11-03

**Authors:** Wenhui Sun, Yifei Li, Shuyang Yu, Jiajie Li

**Affiliations:** 1 School of Transportation and Civil Engineering, Nantong University, Nantong 226019, China; 2 Department of Mechanical Engineering, Huzhou Normal University, Huzhou 313002, China

Concrete and cement-based materials are among the most important engineering materials in modern infrastructure construction. Their mechanical properties, durability, and long-term service performance are closely associated with multiscale factors, including material composition, hydration reactions, pore structure, interfacial characteristics, and internal defects. With increasingly complex service conditions, conventional approaches based mainly on macroscopic experiments and empirical formulations are insufficient to reveal the relationship between internal structural evolution and macroscopic performance. In recent years, advanced characterization techniques, including digital image correlation (DIC), X-ray computed tomography (X-CT), acoustic emission (AE), and microscopic analysis, have been increasingly applied in concrete research, providing effective approaches for quantitatively characterizing crack initiation, localized deformation, and damage evolution [1,2]. Meanwhile, the rapid development of finite element methods, discrete element methods, multiscale modeling approaches, and machine learning techniques has driven a shift in concrete research from empirical descriptions toward mechanistic understanding and quantitative prediction [3]. Furthermore, challenges associated with resource consumption and carbon emissions in the cement and concrete industries have accelerated the development of low-carbon binders, industrial solid wastes, and recycled resources [4]. Therefore, current research on concrete materials is gradually moving toward the integration of advanced characterization, mechanistic understanding, numerical prediction, and data-driven intelligence. The contributions included in this Special Issue, entitled “Advanced Experimental Technology, Theory and Numerical Methods in Concrete Materials,” represent this emerging trend.

Sustainable low-carbon materials and digital manufacturing are important research themes covered in this Special Issue. Various solid wastes, including recycled concrete powder, recycled aggregates, and industrial or biological wastes, have been incorporated into cement-based materials. These studies indicate that waste utilization has evolved from simple material replacement toward the optimization of mechanical performance, microstructure, and environmental sustainability [5,6,7]. Studies on recycled powder activation, rubber modification, aerogel mortar, and geopolymer recycled mortar further show that recycled components can influence not only strength and pore structure, but also thermal properties, deformation capacity, and stability under complex service conditions. Meanwhile, three-dimensional (3D) printing technology has strengthened the connection between material design and manufacturing processes. Extrusion-based printing requires cementitious materials to balance flowability, extrudability, thixotropic recovery, and shape stability [8,9]. The application of various wastes in 3D-printed materials demonstrates that solid wastes can serve not only as replacement materials but also as functional components for rheological regulation and structural build-up. Therefore, future sustainable cement-based materials should focus on the integrated design of material composition, manufacturing processes, engineering performance, and environmental benefits.

Focusing on the mechanical behavior, transport properties, and damage evolution of cement-based materials, this Special Issue highlights the integration of experimental investigations, theoretical analyses, and numerical simulations. Studies on basalt fiber-reinforced concrete, cemented tailings backfill, and leached cement pastes show that variations in macroscopic strength are often a consequence of internal structural evolution. Crack localization, pore structure reconstruction, hydration product transformation, and energy conversion processes are essential for understanding the mechanisms governing material failure and deterioration. Fibers with different lengths can provide synergistic reinforcement through multiscale crack bridging, whereas complex stress disturbances can modify internal damage accumulation and crack coalescence processes. For heat and mass transport in cement-based materials, the combination of hydration microstructure models and multiscale homogenization theories has established connections between microscopic phase compositions and macroscopic transport parameters [10,11]. In fracture investigations, the discrete element method enables direct simulation of crack initiation, propagation, and coalescence, providing complementary insights into internal damage evolution that is difficult to capture experimentally [12,13]. The numerical simulation of concrete with prefabricated array fractures included in this Special Issue further indicates that damage assessment should consider not only crack number but also crack distribution, connectivity, and localization characteristics, which significantly influence the final failure mode. Therefore, future theoretical and numerical studies should strengthen quantitative correlations with experimental observations and establish unified multiscale descriptions linking microstructure, crack evolution, transport processes, and macroscopic properties.

Machine learning and data-driven approaches have provided new opportunities for concrete materials research. With the continuous accumulation of experimental data and engineering databases, neural networks, ensemble learning, and tree-based models have been increasingly applied to predict mechanical strength, volume stability, and structural load-bearing capacity [14,15]. The contributions included in this Special Issue employed Stacking ensemble learning, PSO-BPNN, and Random Search-optimized CatBoost models to predict the compressive strength of geopolymers, shrinkage behavior of self-compacting concrete, and ultimate bearing capacity of concrete-filled steel tubular columns, respectively. These studies indicate that data-driven approaches are gradually evolving from performance fitting toward parameter optimization and model interpretation [16]. However, high prediction accuracy does not necessarily guarantee strong engineering generalization capability. Dataset size, sample distribution, independent validation, and model interpretability remain critical challenges requiring further attention [17]. Recent advances in physics-informed machine learning suggest that incorporating physical laws and mechanical constraints into data-driven models can improve reliability and extrapolation capability under limited data conditions [18]. Therefore, AI should not replace experimental and theoretical approaches, but should be integrated with them to develop physically informed and interpretable prediction frameworks for concrete materials.

Overall, the studies included in this Special Issue demonstrate that research on concrete and cement-based materials is transitioning from conventional single-property testing, empirical analysis, and independent modeling toward integrated multiscale characterization, mechanistic understanding, numerical simulation, and data-driven intelligence. Future research should strengthen the connections between experimental observations and theoretical or numerical models to improve the prediction of crack evolution, transport processes, damage behavior, and long-term service performance. Meanwhile, sustainable material design, digital manufacturing, and artificial intelligence should be incorporated into a more integrated research framework. By integrating experimental observations, theoretical analysis, numerical simulation, and data-driven prediction, a comprehensive framework linking material composition, preparation processes, microstructure, damage evolution, and macroscopic performance can be progressively established. We hope that this Special Issue will promote interdisciplinary interactions among materials science, experimental mechanics, computational mechanics, and artificial intelligence, and contribute to the development of concrete materials with higher performance, lower carbon emissions, enhanced digitalization, and improved intelligence.
